# Supplementary material for: Bacterial precursors and unsaturated long-chain fatty acids are biomarkers of North-Atlantic deep-sea demosponges
Source: PLoS One. 2021 Jan 27;16(1):e0241095. doi: 10.1371/journal.pone.0241095 (PMC7840048; doi:10.1371/journal.pone.0241095)

Fig. S1: Mass spectra of DMDS conducts of C_{26} (a,b) and C_{28} (c,d) LCFA with $\Delta^{9,19}$ (a,c) and $\Delta^{11,21}$ (b,d) unsaturation

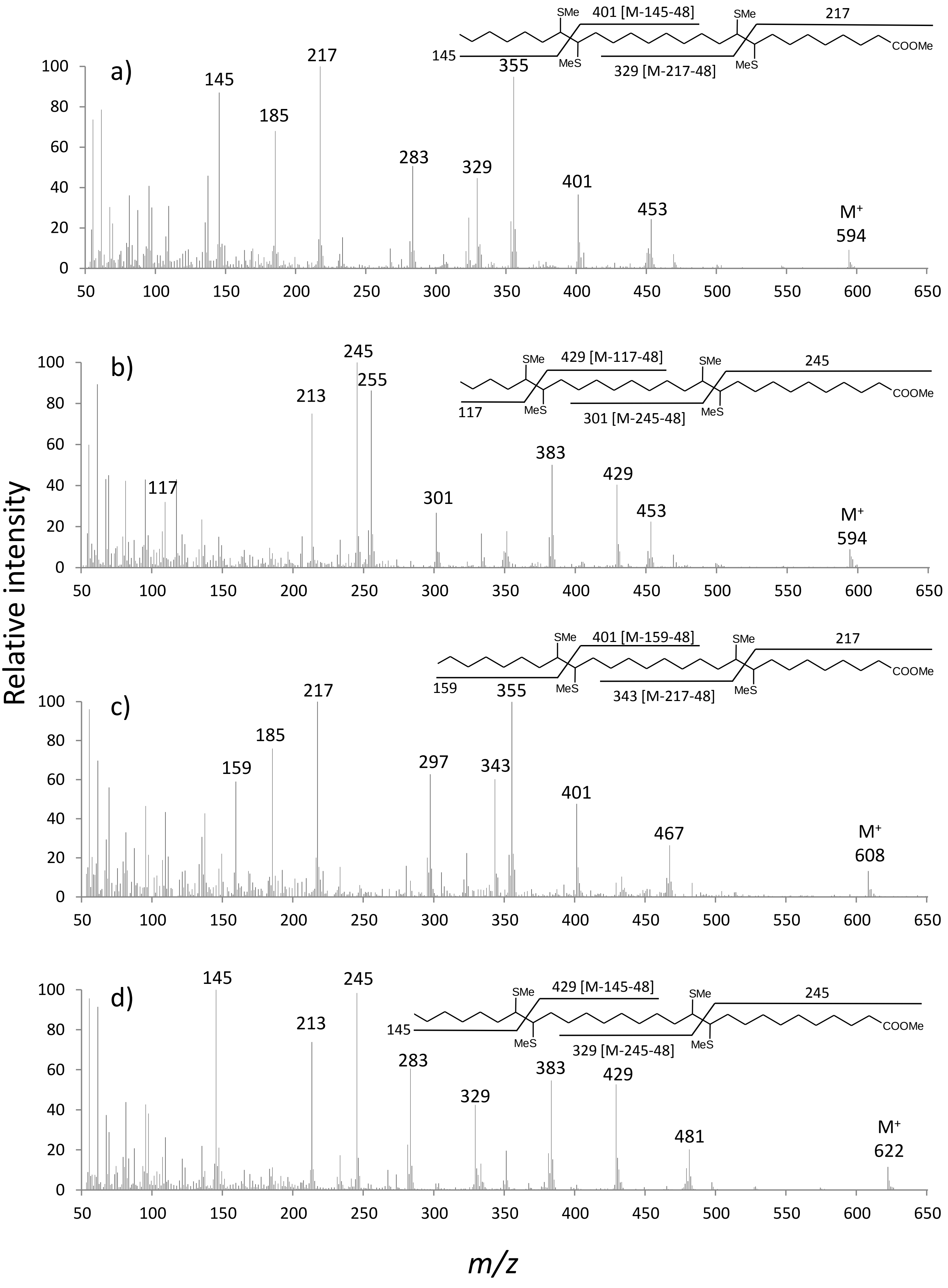

Supplement: S1 Fig — Mass spectra of DMDS conducts of C26 (a,b) and C28 (c,d) LCFA with Δ9,19 (a,c) and Δ11,21 (b,d) unsaturation. (PDF) [file pone.0241095.s001.pdf]
