# Supplementary material for: Bacterial precursors and unsaturated long-chain fatty acids are biomarkers of North-Atlantic deep-sea demosponges
Source: PLoS One. 2021 Jan 27;16(1):e0241095. doi: 10.1371/journal.pone.0241095 (PMC7840048; doi:10.1371/journal.pone.0241095)
Supplement: Attachment — Submitted filename: Coverletter_PlosOne_Response_submitted_v2.docx [file pone.0241095.s003.docx]

| Date  Subject  Reference  Short title | 15-12-2020  Response to reviewers PLOS ONE  PONE-D-20-31393  Fatty acid profiles of deep-sea demosponges | Dr. Anna de Kluijver  Postdoctoral researcher  Department of Earth Sciences  [a.dekluijver@uu.nl](mailto:a.dekluijver@uu.nl)  anna.dekluijver@rvo.nl |
| --- | --- | --- |

Dear Dr. Rodrigues,

We thank the two reviewers for their positive and constructive assessment of our manuscript and we thank you for handling our manuscript. We revised the manuscript according to your and the reviewers’ suggestions and uploaded it with track changes. A point-by-point response is given below.

We hope that we adequately responded to the reviewers comments and thank you for continued consideration.

Yours sincerely,

Anna de Kluijver

**Editorial comments**

*Permits:* No permits were required for field site access in international waters. No special permits were required for Norwegian research expeditions in the Barents Sea region. Both statements have been added to the methodology section.

*Protocol:* The protocol is available online at protocols.io: ([dx.doi.org/10.17504/protocols.io.bhnpj5dn](https://dx.doi.org/10.17504/protocols.io.bhnpj5dn))

*Dual publication.* This submission presents new data that are not part of the publication by Bart et al. (2020). This submission presents the identification, and natural abundance (isotopic) composition of fatty acid profiles of five different demosponge species. For one single species, namely *G. barretti*, we have used the fatty acid profile presented in this submission to study ecological performance (presented in Bart et al. (2020)). As Bart et al. (2020) is a follow-up experimental study, the natural background data for *G. barretti* are presented in this submission. Bart et al. (2020) presents incorporation of deliberately added ^13^C in specific fatty acids to assess the role of symbiotic bacteria in food uptake with no mention of natural abundance composition. Because of the different scope and research question, there is no overlap in tables and figures between our submission and Bart et al., (2020). The profiles of the other 4 species have not been reported before nor are part of Bart et al. (2020).

**Reviewer 1**

*Title:* this is a good suggestion, we added “deep-sea” to the title

*Sponge collection:* Indeed, the precious information on voucher specimens deposition has been added.

*Fame analysis:* We used cold-on-column injection, this has been added.

*Results:* It may not be surprising that the fatty acid (FA) compositions of the phospholipids (PL) fraction and of total lipids are fairly similar, as the majority of fatty acids is present as phospholipids (our study; e.g. Parzanini et al., 2019), although we are aware that glycolipids are also found in sponges (e.g. Bergé and Barnathan, 2005). We cannot exclude that our PL fraction also contained other lipids, such as glycolipids, as was shown by (Heinzelmann et al., 2014). We agree that comparison of fatty acid composition between different lipid classes is interesting, but this was beyond the scope of our study, so we did not investigate this sufficient to justify any conclusions and a comprehensive discussion. However, we added that most fatty acids are present as phospholipids with a reference (Parzanini et al., 2019) and added the quantified PLFA concentrations.

**Reviewer 2**

*Discussion:* Indeed, part of the bacterial fatty acids might be derived from bacterial prey, although their contribution is probably low (Leys et al., 2018). We added this to the discussion.

We also changed FAs to LCFAs in L420 (now L427), as we indeed discuss LFCAs as taxonomic markers.

**References**

Bart MC, de Kluijver A, Hoetjes S, Absalah S, Mueller B, Rapp HT, et al. Differential processing of dissolved and particulate organic matter by deep-sea sponges and their microbial symbionts. Sci Rep. 2020;10:17515**.** <https://doi.org/10.1038/s41598-020-74670-0>

Bergé J-P, Barnathan G. Fatty acids from lipids of marine organisms: molecular biodiversity, roles as biomarkers, biologically active compounds, and economical aspects. Mar Biotechnol. 2005;1:51–111.

Heinzelmann SM, Bale NJ, Hopmans EC, Sinninghe Damsté JS, Schouten S, van der Meer MTJ. Critical assessment of glyco- and phospholipid separation by using silica chromatography. Appl Environ Microbiol. 2014;80(1):360–5.

Parzanini C, Parrish CC, Hamel JF, Mercier A. Functional diversity and nutritional content in a deep-sea faunal assemblage through total lipid, lipid class, and fatty acid analyses. PLoS One. 2018;13(11):1–22.
